# Bilateral Fist Lid-Lift: A Novel Compensatory Behavior in an Infant with Blepharophimosis Syndrome

**DOI:** 10.3390/children13030377

**Published:** 2026-03-06

**Authors:** Biljana Kuzmanović Elabjer, Daliborka Miletić, Mirjana Bjeloš, Mladen Bušić, Iva Bulat, Adrian Elabjer

**Affiliations:** 1 Department of Ophthalmology, Sveti Duh University Hospital, 10000 Zagreb, Croatia; belabjer@kbsd.hr (B.K.E.); dr.mbjelos@gmail.com (M.B.);; 2 Faculty of Dental Medicine and Health Osijek, University Josip Juraj Strossmayer in Osijek, 31000 Osijek, Croatia; busic.iva@gmail.com; 3 Faculty of Medicine Osijek, University Josip Juraj Strossmayer in Osijek, 31000 Osijek, Croatia; 4 School of Medicine, University of Zagreb, 10000 Zagreb, Croatia

**Keywords:** blepharophimosis, ptosis, and epicanthus inversus, blepharoptosis, child development

## Abstract

**Background/Objectives**: To describe a previously unreported compensatory behavior used by an infant with severe bilateral congenital ptosis associated with blepharophimosis syndrome (BPES). **Methods**: Observational case report of a 4.5-month-old infant with severe bilateral congenital upper eyelid ptosis due to BPES. **Results**: The infant demonstrated classic compensatory mechanisms, including frontalis overaction and chin elevation, which were insufficient to clear the visual axis. Notably, she repeatedly used the dorsal surfaces of both fists to elevate the upper eyelids simultaneously and maintain fixation on faces. This behavior ceased following bilateral frontalis suspension surgery with silicone rods. **Conclusions**: In early infancy, severe bilateral ptosis may prompt the emergence of alternative, developmentally constrained compensatory behaviors. The bilateral fist lid-lift appears to represent a visually driven, sensorimotor strategy to clear the visual axis when conventional mechanisms are ineffective. Recognition of this behavior expands understanding of early compensatory responses in congenital ptosis and BPES.

## 1. Introduction

Blepharophimosis syndrome or blepharophimosis–ptosis–epicanthus inversus syndrome (BPES) is a rare congenital disorder characterized by a tetrad of distinctive ocular anomalies: blepharophimosis, severe bilateral ptosis, epicanthus inversus, and telecanthus [1]. Among these features, congenital ptosis represents the most clinically significant abnormality, frequently resulting in severe visual impairment and amblyopia. In BPES, ptosis is typically severe and bilateral, with minimal or absent levator function.

The degree of eyelid drooping often necessitates adaptive compensatory mechanisms aimed at preserving visual input. These strategies evolve in parallel with neurological and motor maturation. During early infancy, compensation is limited and primarily achieved through axial mechanisms, most notably a persistent chin-up head posture, as well as progressive recruitment of the frontalis muscle [1,2]. Manual elevation of the upper eyelids represents a later-developing compensatory strategy, as it requires the maturation of voluntary fine motor control, coordinated hand-to-face movements, visuomotor integration, and cognitive awareness of visual obstruction [2]. For this reason, intentional manual eyelid lifting is generally expected to appear in the second half of the first year of life, when purposeful grasping and targeted motor actions become established.

Here, we present a previously unreported case of early-onset compensatory manual eyelid elevation in an infant with severe bilateral congenital ptosis associated with BPES, expanding current understanding of adaptive motor responses in the context of profound congenital visual axis obstruction.

## 2. Case Presentation

A 4.5-month-old female infant with BPES was referred to our Clinic with severe bilateral congenital upper eyelid ptosis. The condition was hereditary; her father and paternal cousin also had BPES. Out of four siblings born to the same parents, this infant was the only one diagnosed with the condition. She was delivered via cesarean section because of breech presentation. At birth, additional findings included microcephaly with a thin corpus callosum, micrognathia, and an omega-shaped epiglottis associated with laryngomalacia.

On examination, the infant showed classic BPES features. She had blepharophimosis with a horizontal palpebral aperture of 15 mm and severe upper eyelid ptosis with less than 2 mm of vertical opening. Epicanthus inversus was also present. Other facial findings included absent upper eyelid skin crease, telecanthus (intercanthal distance 25 mm), low nasal bridge, and low-set ears. Forced eyelid opening revealed bilateral jerk nystagmus, but the rest of the anterior and posterior segment exams were unremarkable.

**Figure 1:**
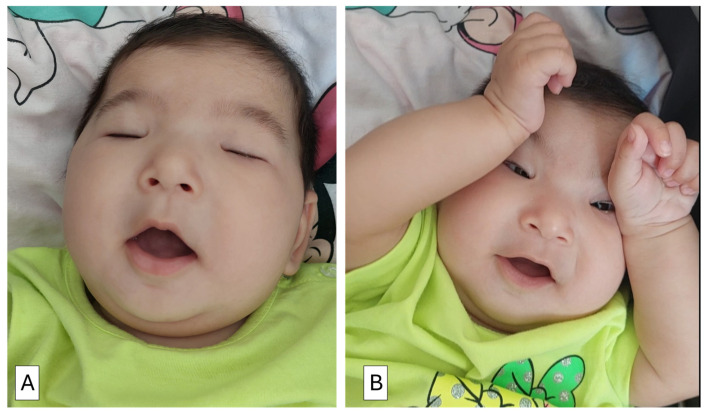
(**A**) A 4.5-month-old girl with blepharophimosis syndrome demonstrating severe bilateral congenital ptosis. Frontalis overaction and chin elevation are present but insufficient to clear the visual axis; (**B**) The infant elevates both upper eyelids simultaneously using both fists while orienting toward the examiner’s face. Sustained lift allows maintenance of fixation during face-to-face interaction.

In response to mechanical obstruction of the visual axis, the infant demonstrated commonly observed compensatory postures, including frontalis overaction and chin elevation. These actions were not enough to open her eyes (Figure 1A). Most strikingly, she instinctively brought both fists to her eyelids, using them to lift both lids at once and holding them in place as she focused on the examiner’s face (Figure 1B, Video S1).

**Figure 2:**
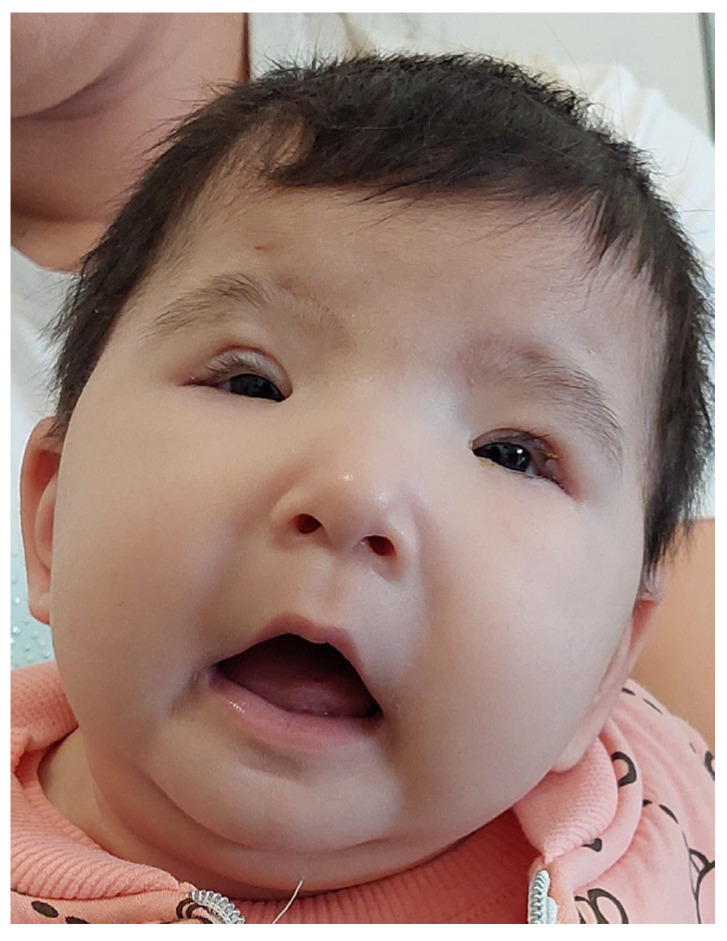
At five months old, postoperative appearance following bilateral frontalis suspension with silicone rods. The visual axis is cleared, and the fist lid-lift behavior is no longer observed.

At five months of age, the infant underwent bilateral upper eyelid ptosis correction with frontalis suspension using a silicone rod. The surgical procedure was uneventful, and during the postoperative period, the “bilateral fist lid-lift” behavior ceased (Figure 2).

## 3. Discussion

In severe bilateral congenital ptosis associated with BPES, the infant’s ability to perceive faces and other important visual cues is markedly restricted. Because early social and visual development depends heavily on face recognition and high-contrast stimuli, obstruction of the visual axis substantially alters sensory experience. When exposed to salient stimuli (such as the voice of the carer, a novel sound, or close facial proximity) infants demonstrate a strong orienting response and attempt to fixate [2,3,4]. In the presence of severe ptosis, this creates increased motivation to clear the visual axis. A previous report described an infant with BPES who was able to elevate the eyelids only by using the thumbs [5]. Other references note that children may mechanically lift a ptotic eyelid with a finger as a compensatory behavior; however, these descriptions lack precise age documentation or present the behavior as a general observation rather than as a structured developmental phenomenon [6,7]. To our knowledge, a bilateral fist-based lid elevation used to maintain fixation in early infancy has not previously been described within an ontogenetic framework.

In our case, typical compensatory strategies, namely frontalis overaction and chin elevation, were present but insufficient to clear the visual axis adequately. This suggests that when primary axial and facial compensatory mechanisms fail, infants with severe ptosis may generate alternative solutions. Compensatory behaviors in congenital ptosis likely emerge sequentially. Chin elevation may appear as early as 1–3 months of age, coinciding with improving cervical control and early visual-vestibular integration [8]. These visually driven postural adaptations precede higher-level voluntary problem-solving and support the hypothesis that an early sensorimotor, fist-based strategy can arise before refined manual control develops. With further maturation of facial musculature and visuomotor coordination, typically between 6 and 9 months, infants increasingly recruit the frontalis muscle more effectively [4]. Purposeful manual lifting behaviors develop later. Between 4 and 6 months, infants acquire voluntary reaching and grasping; however, hand-to-face contact remains largely exploratory rather than functionally compensatory. Between 6 and 9 months, coinciding with stable sitting and improved eye–hand coordination, children with significant ptosis may begin to demonstrate intentional pulling of the upper eyelids or brows while attempting to fixate on objects of interest. By 9 to 12 months, with refinement of radial grasp and early pincer grip, manual elevation of the eyelids can become a reproducible and situational compensatory behavior [2,3].

The use of fists to elevate the upper eyelids in this 4.5-month-old infant reflects the neurodevelopmental constraints characteristic of this age. At this stage, subcortical and brainstem mechanisms likely drive this pattern, using primitive reflexes and visual and auditory orienting networks. A plausible sensorimotor “clear-the-visual-axis” loop may operate as follows: the infant orients toward a salient stimulus, brings the hand to the face through a reflex-biased motor pathway, and incidentally elevates the eyelids. Partial restoration of vision, particularly the sudden perception of a face or high-contrast object, provides immediate sensory reinforcement. This rapid perceptual reward strengthens repetition of the behavior, even in the absence of conscious intent [9,10].

Hand-to-face coupling is well documented in early infancy and is closely linked to primitive reflexes associated with feeding and self-regulation, such as the rooting reflex [11]. Although the rooting reflex typically diminishes by 4–6 months, residual circuitry may continue to bias hand movements toward the perioral and facial region during stimulation. Motor development at this age remains characterized by limited fine motor differentiation. Bimanual movements are common in early infancy and are generally attributed to limited hemispheric specialization, immature interhemispheric inhibition, and the predominance of midline-symmetric motor programs at this developmental stage. In the present case, thinning of the corpus callosum (an essential structure for interhemispheric connectivity) was also observed, which may further predispose to symmetric motor output; however, this association remains speculative. Early reaching is frequently accompanied by closed fist posture. Although anticipatory hand opening begins to improve around four months of age, it remains inconsistent [11]. The palmar grasp reflex and whole-hand dominance may persist until approximately six months, reflecting ongoing cortical maturation and gradual emergence of voluntary, individuated finger movements [12].

Within this developmental context, the fist represents the most accessible and biomechanically stable tool available. It provides a broad, firm contact area capable of elevating the eyelids without requiring fine motor precision. This approach offers strong tactile and proprioceptive feedback and likely reduces the risk of inadvertent ocular injury compared with the use of an uncoordinated single finger. As manual skills and cortical control mature, infants develop more refined and purposeful techniques, such as using individual fingers to lift the eyelids [5].

The strongest evidence that the “bilateral fist lid-lift” constitutes a functional compensatory mechanism rather than a motor stereotypy is its complete disappearance following frontalis suspension surgery. Surgical restoration of a stable visual axis resulted in immediate cessation of the behavior, supporting a direct causal relationship between visual deprivation and the observed compensatory pattern.

## 4. Conclusions

In contrast to the expected developmental timeline—where purposeful manual lifting of the eyelids typically emerges only after the maturation of voluntary grasping and visuomotor coordination in the second half of the first year of life—this patient demonstrated hand-assisted eyelid elevation at a markedly younger age. The behavior was reproducible, visually goal-directed, and clearly associated with attempts to improve fixation, suggesting an accelerated adaptive response to significant visual axis obstruction.

To our knowledge, such early manifestation of manual compensatory activity has not been previously described in the context of BPES-related ptosis. This observation expands the current understanding of adaptive strategies in infants with severe congenital ptosis and highlights the potential for earlier-than-expected integration of visual need and motor output. Recognition of this phenomenon may have important implications for clinical assessment, developmental interpretation, and the timing of surgical intervention in affected patients.

## Data Availability

The original contributions presented in this study are included in the article. Further inquiries can be directed to the corresponding author.

## Supplementary Materials

The following supporting information can be downloaded at: https://www.mdpi.com/article/10.3390/children13030377/s1, Video S1: bilateral fist lid-lift.

## Abbreviations

The following abbreviation is used in this manuscript:

BPES	Blepharophimosis-Ptosis-Epicanthus Inversus Syndrome or Blepharophimosis Syndrome

## References

[1] Neuhouser A.J., Zeppieri M., Harrison A.R. (2025). Blepharophimosis Syndrome. StatPearls.

[2] Atkinson J. (2000). The Developing Visual Brain.

[3] Wright K.W., Spiegel P.H., Thompson L.S. (2006). Handbook of Pediatric Strabismus and Amblyopia.

[4] Chinn L.K., Noonan C.F., Hoffmann M., Lockman J.J. (2019). Development of Infant Reaching Strategies to Tactile Targets on the Face. Front. Psychol..

[5] Noda K., Mashima Y., Nakamura Y., Tanaka Y. (1998). Blepharophimosis-ptosis-epicanthus inversus syndrome associated with interstitial deletion of chromosome 3q21-23. J. Pediatr. Ophthalmol. Strabismus.

[6] Finsterer J. (2003). Ptosis: Causes, presentation, and management. Aesthetic Plast. Surg..

[7] Sudhakar P., Vu Q., Kosoko-Lasaki O., Palmer M. (2009). Upper Eyelid Ptosis Revisited. Am. J. Clin. Med..

[8] Boricean I.D., Bărar A. (2011). Understanding ocular torticollis in children. Oftalmologia.

[9] Johnson M.H. (2005). Subcortical face processing. Nat. Rev. Neurosci..

[10] Di Paolo E.A., Barandiaran X.E., Beaton M., Buhrmann T. (2014). Learning to perceive in the sensorimotor approach: Piaget’s theory of equilibration interpreted dynamically. Front. Hum. Neurosci..

[11] Addabbo M., Roberti E., Colombo L., Picciolini O., Turati C. (2022). Newborns’ early attuning to hand-to-mouth coordinated actions. Dev. Sci..

[12] Futagi Y., Toribe Y., Suzuki Y. (2012). The grasp reflex and moro reflex in infants: Hierarchy of primitive reflex responses. Int. J. Pediatr..

